# Suppression of *Plasmodium falciparum *by serum collected from a case of *Plasmodium vivax *infection

**DOI:** 10.1186/1475-2875-7-113

**Published:** 2008-06-26

**Authors:** Yoshiro Nagao, Masako Kimura-Sato, Porntip Chavalitshewinkoon-Petmitr, Supatra Thongrungkiat, Polrat Wilairatana, Takafumi Ishida, Peerapan Tan-ariya, J Brian de Souza, Srivicha Krudsood, Sornchai Looareesuwan

**Affiliations:** 1 Faculty of Tropical Medicine, Mahidol University,420/6 Rajvithi Road, Bangkok, 10400, Thailand; 2 Department of Biological Sciences, Graduate School of Science, the University of Tokyo, Hongo 3, Bunkyo, Tokyo, Japan; 3 Department of Microbiology, Faculty of Science, Mahidol University, Rama 6 Road, Bangkok 10400, Thailand; 4 Department of Infectious and Tropical Diseases, London School of Hygiene and Tropical Medicine, Keppel Street, London WC1E 7HT, UK

## Abstract

**Background:**

It has frequently been reported that *Plasmodium vivax *suppressed *Plasmodium falciparum *and ameliorated disease severity in patients infected with these two species simultaneously. The authors investigate the hypothesis that immunological responses stimulated by *P. vivax *may play a role in suppressing co-infecting *P. falciparum*.

**Methods:**

Sera, taken sequentially from one of the authors (YN) during experimental infection with *P. vivax*, were added to *in vitro *cultures of *P. falciparum*. Cross-reactive antibodies against *P. falciparum *antigens, and cytokines were measured in the sera.

**Results:**

Significant growth inhibitory effects upon *P. falciparum *cultures (maximally 68% inhibition as compared to pre-illness average) were observed in the sera collected during an acute episode. Such inhibitory effects showed a strong positive temporal correlation with cross-reactive antibodies, especially IgM against *P. falciparum *schizont extract and, to a lesser degree, IgM against Merozoite Surface Protein (MSP)-1_19_. Interleukin (IL)-12 showed the highest temporal correlation with *P. vivax *parasitaemia and with body temperatures in the volunteer.

**Conclusion:**

These results suggest the involvement by cross-reactive antibodies, especially IgM, in the interplay between plasmodial species. IL-12 may be one of direct mediators of fever induction by rupturing *P. vivax *schizonts, at least in some subjects. Future studies, preferably of epidemiological design, to reveal the association between cross-reactive IgM and cross-plasmodial interaction, are warranted.

## Background

A number of studies have reported that *Plasmodium *species apparently suppressed each other, in population [1,2], or in a mixedly-infected individual [3-8]. It is noteworthy that the emergence of *Plasmodium vivax *in patients' peripheral blood has led to a "total disappearance" of *Plasmodium falciparum*, while passive transfer of *P. falciparum*-immune IgG exhibited weaker suppressive effects on *P. falciparum *in these patients [9]. The severity of *P. falciparum *infection has been reported to be dramatically ameliorated in patients simultaneously infected with *P. vivax *[2,10]. Mutual suppression between *Plasmodium *parasites has been thoroughly reviewed [11-13]. These observations have led to speculation that the much lower disease-specific mortality and case fatality rate from malaria in Asia-Pacific region than in Africa may be due to the presence of so-called "benign" *P. vivax *malaria [14,15], because *P. falciparum *is by far the most dominant in sub-Saharan Africa [16]. Therefore, understanding the interaction of these *Plasmodium *species is important, especially because several *P. vivax *vaccines are currently being developed. The authors hypothesized that the host immunological reactions to infecting *P. vivax *might be playing a role in this suppression of co-infecting *P. falciparum*. Although such a notion has been long held [17-19], actual data has been rarely available for human malaria. However, any study design that leaves ailing human volunteers (other than the researcher) untreated is unacceptable from a bioethical point of view. By contrast, "almost all of the associated legal, ethical, and metaphysical problems vanish" with self-recruitment of the researcher, according to the founder of modern bioethics [20]. Hence, to examine the hypothesis, YN volunteered to be infected with *P. vivax*, and his serum was collected sequentially during this infection. The inhibitory effect of these sera upon *P. falciparum *growth was quantified by using an *in vitro *culture technique. To illustrate the kinetics of the immunological processes, immunological mediators including cross-reactive antibodies against *P. falciparum *and cytokines in these sera were also measured.

## Case presentation

### Human subjects

The volunteer (YN, an author) was a 35-year old Japanese man. He had previously experienced *Plasmodium *infection only once, when he was infected with *P. vivax*, one year before the present study [21]. Control subjects were composed of 21 healthy adult Bangkok residents (9 male and 12 female, median age: 33 years) who had never been infected with any *Plasmodium *species.

### *Plasmodium vivax *parasites

In January 2001, 2 ml of whole blood was obtained from an adult patient who was infected with *P. vivax *near Thailand-Myanmar border. PCR confirmed that this blood was infected with *P. vivax *but not with other *Plasmodium *species [22]. Fifty female anopheline mosquitoes were fed on this blood in a membrane feeder. One month later, sporozoites were detected in the salivary glands of two randomly selected mosquitoes.

### Course of illness in the volunteer

The volunteer was challenged by another infected mosquito on day 0. The mosquito was tested by PCR, confirming the presence of *P. vivax *only. The volunteer was continuously monitored in Mahidol University Hospital for Tropical Diseases in Bangkok, a non-malarious region. The volunteer experienced microscopically positive parasitaemias of *P. vivax *from day 14. From day 20, a regimen of artemisinin (200 mg/day for 3 days) and doxycycline (100 mg/day for 6 days) was administered to radically terminate the blood-stage parasites. The blood collection was resumed on day 29, when the anti-malarial drugs should have been completely eliminated from the volunteer's blood [23,24]. Subsequently YN relapsed, and parasites were detected in the peripheral blood microscopically on day 36. After experiencing an acute phase, he was treated with chloroquine (1,500 mg on day 52; 500 mg on day 53 and 54), and with primaquine (15 mg per day for 14 days) to radically kill both the blood-stage parasites and the hypnozoites. The illness, especially headache, was subjectively much milder than in the previous infection one year before; nevertheless, the peak parasitaemia was three-times higher than the previous one.

### Blood collection

Venous blood was collected every day as follows: (i) the pre-challenge period (days -6 to 0); (ii) the initial infection period (days 1 to 20); (iii) the relapse period (days 29 to 53). Blood was collected 7, 29 and 29 times during the periods (i), (ii) and (iii), respectively. The sera collected during the period (i) provided the baseline. The volunteer was phlebotomized once per day when the body temperature did not show a noticeable elevation; otherwise twice per day (one at a peak of body temperature and one at about 12 hours after the peak). Blood was allowed to coagulate at 25°C for 20 minutes, and centrifuged. Serum was preserved at -70°C until use. No drugs were administered from day -20 and throughout the entire period of the infection, except for the above medications.

### *In vitro *cultures of *Plasmodium falciparum*

**Figure 1:**
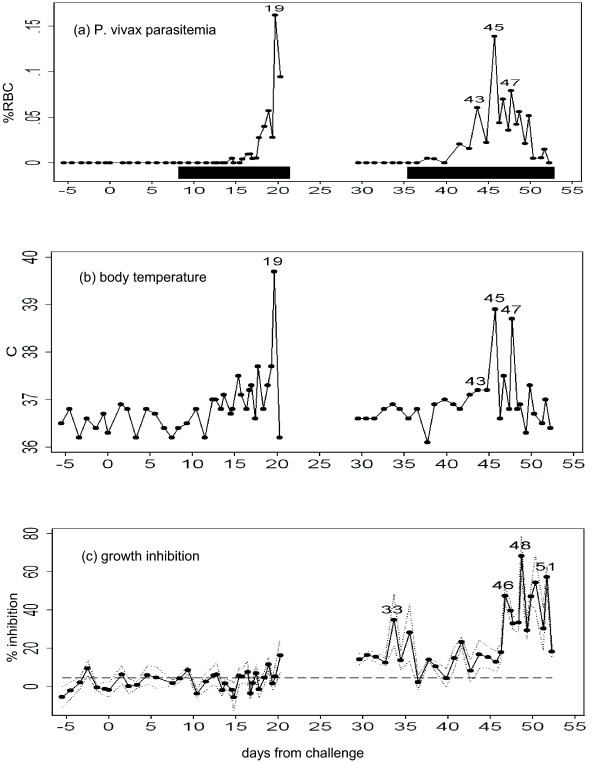
**Course of illness in the volunteer and *in vitro *growth inhibition on *P. falciparum *exhibited by the volunteer's sera**. (a) *Plasmodium vivax *parasitaemias (percentage of infected red blood cells) determined microscopically in the volunteer are represented by a line, while detection of *P. vivax *by PCR is denoted by horizontal black bars. (b) Body temperatures measured at the time of phlebotomies are shown. (c) The solid line represents the average of *in vitro *growth inhibition obtained from four cultures, while the dotted lines represent standard errors. The broken line denotes the upper 95% confidence limit of the baseline sera. Days of high values are denoted on the symbols in this and subsequent figures. Day 0 is the day of challenge.

*Plasmodium falciparum *was cultured *in vitro*, in each of the volunteer's sera, as follows. K1 strain of *P. falciparum *was synchronized into the ring form stage [25], and subsequently mixed with the incomplete medium and the purified type-O red blood cells, so that the *P. falciparum *parasitaemia was set to 0.5% and the haematocrit was adjusted to 50%. In the each well on a 96-well flat-bottomed plate, 10 μl of this parasite preparation, 45 μl of the incomplete medium, and 35 μl of each of the volunteer's sera were mixed (final volume 90 μl; hematcrit 5.6%; serum concentration 39% (v/v)). The plate was incubated under 5% CO_2_pressure at 37°C. After 24 hours, 60 μl of the supernatant was replaced by 35 μl of the the same serum and 45 μl of incomplete medium. After a further 28 hours, two thin films were prepared from each well. The percent reduction from the baseline was defined as the growth inhibition of the each serum. As a result, the volunteer's sera collected during the relapse period exhibited growth inhibitory effects, significantly elevated above the baseline level (Figure 1c). This was particularly marked between days 46 and 51 (mean: 42%, maximum: 68%), and to a lesser degree, between days 33 and 35.

### Measurement of cross-reactive antibodies

**Figure 2:**
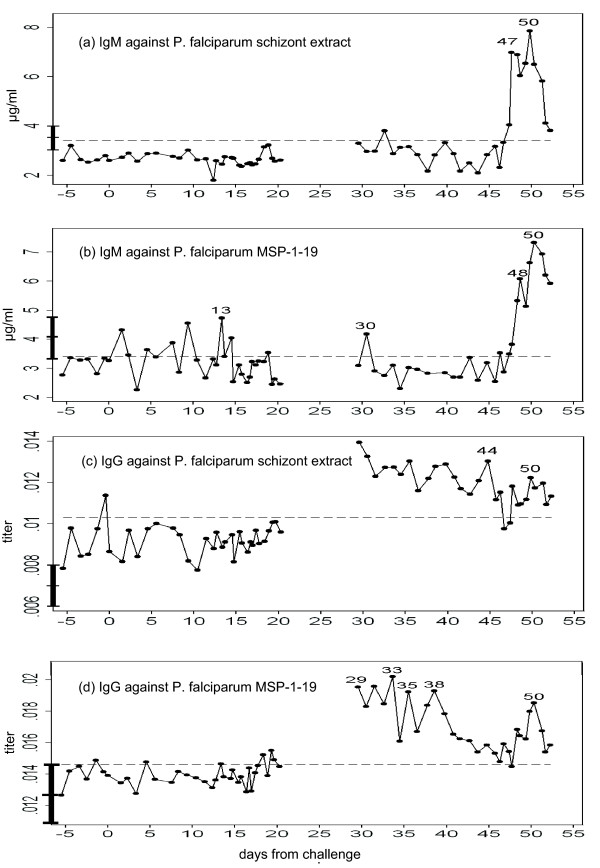
**Cross-reactive antibodies against *Plasmodium falciparum *antigens**. (a) IgM against *P. falciparum *schizont extract, (b) IgM against *P. falciparum *Merozoite Surface Protein (MSP)-1_19_, (c) IgG against *P. falciparum *schizont extract, and (d) IgG against *P. falciparum *MSP-1_19_. The solid line represents the concentration of IgM, or the titer of IgG as compared to the hyper-immune pooled serum (titer = 1). The broken line denotes the upper 95% confidence limit obtained from the baseline, while the mean and confidence interval in the control subjects are denoted as the vertical bar overlapping Y axis.

To investigate the degree of cross-reactive immunity in the sera, we measured IgM and IgG levels against *P. falciparum *schizont extract and Merozoite Surface Protein (MSP)-1_19 _of *P. falciparum*. ELISA was carried out as previously described [26]. *P. falciparum *schizont extract and MSP-1_19_(Wellcome sequence) were obtained as described in [26-28]. Each IgG plate contained a standard dilution series of a laboratory reference hyper-immune adult Gambian plasma pool. For IgM, a standard dilution series of non-specific IgM purified from human serum (Sigma-Aldrich) was coated in one set of wells. Of note, IgM values against *P. falciparum *schizont extract and MSP-1_19 _were elevated after day 47 (Figure 2a,b). The temporal correlation between pairs of time-series variables, such as growth inhibitory effect and antibody level, were quantified as follows. First, simple linear regression was applied. Next, since the blood was sampled at non-random time points and temporally non-independent, we also employed Prais-Winsten's method for time-series regression [29]. In doing so, the time-axis was divided into discrete time-steps of a fixed length (half day, one day and two days). When a time-step included more than one sample, the averaged value was used. As a result, the *in vitro *growth inhibitory effects on *P. falciparum *and the levels of IgM against *P. falciparum *(especially, schizont extract) showed strong temporal correlation, in any time-step lengths examined (Table 1).

**Table 1:** Results of analyses which regressed *in vitro Plasmodium falciparum *growth inhibition against each cross-reactive antibody level.

	Simple linear regression	Prais-Winsten's regression		
		half-day^††^	one-day^††^	two-day^††^
	(N = 64^†^)	(N = 62)	(N = 50)	(N = 27)
IgM against schizont extract	R^2 ^= 0.50 (P < 0.0001)	R^2 ^= 0.43 (P < 0.0001)	R^2 ^= 0.44 (P < 0.0001)	R^2 ^= 0.56 (P < 0.0001)
IgM against MSP-1_19_	R^2 ^= 0.38 (P < 0.0010)	R^2 ^= 0.16 (P = 0.0016)	R^2 ^= 0.18 (P = 0.0022)	R^2 ^= 0.16 (P = 0.036)
IgG against szhitont extract	R^2 ^= 0.22 (P = 0.0001)	R^2 ^= 0.011 (P = 0.41)	R^2 ^= 0.026 (P = 0.27)	R^2^not obtained (P = 1.0)
IgG against MSP-1_19_	R^2 ^= 0.029 (P = 0.18)	R^2 ^= 0.079 (P = 0.063)	R^2 ^= 0.22 (P = 0.0006)	R^2 ^= 0.070 (P = 0.18)

Each regression analysis was conducted against single independent variable. ^†^One serum sample (day 6) was not available for antibody measurements and growth inhibition assays. ^†† ^For Prais-Winsten's analysis, the data was aggregated in each time-step of half-day, one-day or two-day lengths.

### Cytokines

**Figure 3:**
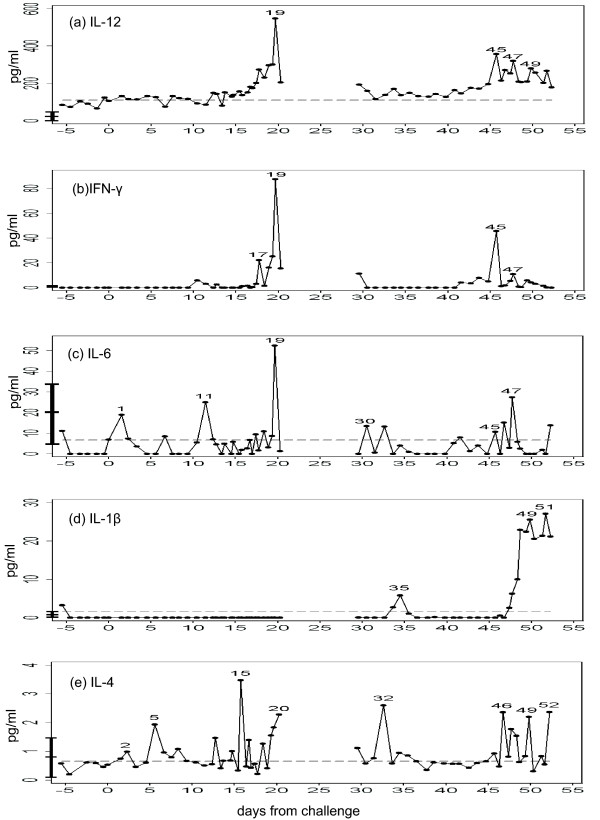
**Serum cytokine levels**. Concentrations of (a) IL-12, (b) IFN-γ, (c) IL-6, (d) IL-1β, (e) IL-4 (all in pg/ml) (solid lines). The broken line denotes the upper limit of 95% confidence interval obtained from the baseline, while the mean and confidence interval in the control subjects are shown as the vertical bar overlapping Y axis.

Interferon (INF) -γ, Interleukin (IL) -1β, IL-2, IL-4, IL-6, IL-12p40, TNF-α were measured, at least in duplicate, using commercial ELISA kits (Diaclone, Besancon). TNF-α showed highly variable values among multiple measurements (data not shown). Among the cytokines examined, IL-12 showed the highest correlation with *P. vivax *parasitaemia (compare Figure 3a vs Figure 1a). When IL-12 was regressed against the parasitaemia, Prais-Winsten's R^2 ^at half-day time-steps was 0.62 (P < 0.0001). This suggested that IL-12 is closely associated with schizont rupture. IFN-γ was extremely spiky and appeared only in the first few paroxysms (defined as a body temperature above 37.5°C), both in initial and relapse periods (Figure 3b). Other cytokines showed weaker association with paroxysm or parasitaemia (Figure 3c,d,e).

## Conclusion

Host antibody responses against *Plasmodium *are heterogeneous across different ethnicities [30], ages and accumulated exposure [31]. A single observation from one volunteer cannot, therefore, be generalized. However, despite this caveat, the present study suggests the possibility that cross-reactive antibodies, especially IgM, in an individual who has been infected only with *P. vivax *suppress growth of *P. falciparum*. Although IgM does not undergo a process of affinity maturation, this is compensated for by the fact that it is a pentamer. At least one report found that IgM exhibited a stronger neutralizing effects on *Plasmodial *merozoites than did IgG [32]. Indeed, this lower specificity may make IgM more likely show cross-species reactivity. The short-lived nature of IgM may also explain why mutual suppression across *Plasmodial *species is rather transient [6] (also reviewed in [33]), or why immunization with one species rarely protected the vaccinees from other species [34]. *In vivo*, IgG-mediated cellular mechanisms [9,35], may also play an important role in the cross-*Plasmodium *interaction.

IL-12, which showed the highest correlation with *P. vivax *parasitaemia, is hence likely to be (one of) the most direct mediator(s) bridging from rupturing schizonts to fevers. It was reported that IL-12 and IFN-γ synergistically enhance the parasiticidal activities of peripheral blood mononuclear cells [36-41]. Therefore, the sharp peaks of INF-γ under the presence of IL-12 observed in the volunteer could mount an early *in vivo *defense against blood-stage parasites, and may contribute to the suppressive effect of *P. vivax *on *P. falciparum*.

Collectively, these results suggest a possibility that *P. vivax *infections may suppress *P. falciparum *in multiple ways including cross-reactive IgM and cytotoxicity-inducing cytokines. To thoroughly prove that *Plasmodium*-specific IgM is playing a major role in cross-*Plasmodium *suppression, such specific IgM should have been purified. Such studies should be part of any further work to test the hypothesis proposed here, preferably within an epidemiologically appropriate framework.

## Consent

The present study was approved by the Committee for Ethics, Faculty of Tropical Medicine, Mahidol University. Written informed consent was obtained from the patient for publication of this case report and any accompanying information.

## List of abbreviations used

MSP: Merozoite Surface Protein; IL: Interleukin.

## Competing interests

The authors declare that they have no competing interests.

## Authors' contributions

YN conceived this study, and asked other authors to participate in this study, MKS assumed responsibility for PCR, PCP and PT assisted in *P. falciparum *cultures, ST prepared the infected mosquitoes, SK and PW assumed the responsibility for the clinical management of YN, JBS assisted in immunological measurements, TI assisted in PCR and measurements of cytokines, SL organized the entire study. All authors read and approved the final manuscript.
